# Analysis of Key Control Points of Microbial Contamination Risk in Pork Production Processes Using a Quantitative Exposure Assessment Model

**DOI:** 10.3389/fmicb.2022.828279

**Published:** 2022-03-24

**Authors:** Tengteng Yang, Ge Zhao, Yunzhe Liu, Lin Wang, Yubin Gao, Jianmei Zhao, Na Liu, Xiumei Huang, Qingqing Zhang, Junhui Liu, Xiyue Zhang, Junwei Wang, Ying Xu

**Affiliations:** ^1^Laboratory of Pathogenic Microorganisms Inspection, Livestock and Poultry Products Quality & Safety Risk Assessment Laboratory (Qingdao) of MARA, China Animal Health and Epidemiology Center, Qingdao, China; ^2^College of Food Science and Engineering, Ocean University of China, Qingdao, China

**Keywords:** swine-slaughtering processes, microbial contamination risk, quantitative exposure assessment model, key control points, multilocus sequence typing

## Abstract

Pork is one of the most common foods causing microbial foodborne diseases. Since pork directly enters the market after slaughtering, the control of microbial contamination in the slaughtering processes is the key to ensuring the quality and safety of pork. The contamination level of *Escherichia coli*, a health-indicator bacterium, can reflect the risk level of potential pathogens. In order to assess the *E. coli* exposure risk of pork during slaughtering and to identify the key control points, we established an *E. coli* quantitative exposure assessment model for swine-slaughtering processes in slaughterhouses of different sizes. The model simulation data indicated the *E. coli* contamination pattern on the surfaces of swine carcasses during slaughtering. The changes in *E. coli* contamination were analyzed according to the simulation data of each slaughtering process. It was found that the number of *E. coli* after trimming in big and small slaughterhouses increased to the maximum values for the whole processes, which were 3.63 and 3.52 log_10_ CFU/100 cm^2^, respectively. The risk contribution of each slaughtering process to the *E. coli* contamination on the surface of terminal swine carcasses can be determined by correlation analysis. Because the absolute value of correlation coefficient during the trimming process was maximum (0.49), it was regarded as the most important key control point. This result can be further proved via the multilocus sequence typing of *E. coli*. The dominant sequence type before trimming processes was ST10. ST1434 began to appear in the trimming process and then became the dominant sequence type in the trimming and pre-cooling processes. The model can provide a theoretical basis for microbial hygiene supervision and risk control in swine-slaughtering processes.

## Introduction

Pork has played a dominant role in China’s meat consumption for a long time, accounting for more than 60% of the total meat consumption since 2000 (Min et al., 2015). Pork directly enters the market after slaughtering, and it is directly exposed to the surrounding environment from the beginning of the slaughtering process. Since the microbial contamination caused by the devices and appliances used for slaughtering is inevitable (Van Ba et al., 2019), the hygienic control of the slaughtering processes is indispensable. Some scholars pointed out that the number of microorganisms on the carcass surface after slaughtering has a significant impact on the shelf life and safety of products (Gill et al., 1998). Therefore, controlling microbial contamination during slaughtering processes is the key to ensure the quality and safety of meat.

*Escherichia coli* is an intestinal symbiotic bacterium in humans and animals, and most of its strains are harmless to people. In the case of poor environmental sanitation, it is often scattered in the surrounding environment with feces. Because there are some normal flora in addition to intestinal pathogenic bacteria in the feces, *E. coli* can be used as a health-indicator bacterium to reflect food hygiene and the risk to human health. The detection of *E. coli* indicates that there may be fecal contamination. Repeated detection of *E. coli* in multiple processes of food production indicates that the risk of food safety is increasing, and the risk of possible cross-contamination of various pathogenic microorganisms is high (Kusturov et al., 2017).

Key control points are important steps or processes determined by analyzing raw materials, production processes, and human factors affecting product quality and safety (Setiabuhdi et al., 1997). Analysis of key control points can control or eliminate food safety hazards to acceptable levels. Quantitative microbiological risk assessment (QMRA) has been increasingly investigated and paid more attention in food safety management and control worldwide, including China (Dong et al., 2015; Wu et al., 2018). As an important part of risk assessment, exposure assessments can provide useful information for identifying key control points. An exposure assessment model is a more scientific assessment method based on monitoring data, which is formed by data fitting, logical operation, and random sampling simulation. In order to explore the key control points affecting the risk of microbial contamination in pork during slaughtering more scientifically, it can be realized by establishing an *E. coli* quantitative exposure assessment model in different swine-slaughtering processes. Some studies have aimed at establishing microbial risk assessment models for the “farm-to-fork” food chain or retail and subsequent links (Dang-Xuan et al., 2018; Zhang et al., 2018). In addition, because pork has been identified as one of the main sources of *Salmonella* infection in humans, the risk assessment of microorganisms in pork also focused on *Salmonella* (Swart et al., 2016; Gurman et al., 2018). However, there are other unknown risks posed by pathogenic microorganisms in pork. Insufficient understanding of the application of health-indicator bacteria in exposure assessment and the difficulties of sample collection in swine-slaughtering lines hinder the establishment of such an exposure assessment model. Therefore, exposure assessment of health-indicator bacteria such as *E. coli* used to analyze the overall contamination risk of pathogenic microorganisms during swine-slaughtering is urgently needed.

In this study, an *E. coli* exposure assessment model suitable for China’s general swine-slaughtering processes was established, and the key control points of microbial contamination were analyzed. Our study provides a scientific basis for effectively controlling the risk of pathogenic microorganisms and improving the quality of pork products.

## Materials and Methods

### Data Information During Swine Slaughtering

#### Sample Collection

All contamination data of *E. coli* from various sources in the slaughterhouses were from the monitoring data of our laboratory. From August to September 2020, we chose five swine slaughterhouses of different sizes in Shandong Province, including three large slaughterhouses and two small slaughterhouses. Large slaughterhouses slaughter more than 300 swine per hour, and small slaughterhouses slaughter between 30 and 70 swine per hour (GB 50317-2009, 2009). We collected 359 surface samples of swine carcasses and 139 environmental samples from six slaughtering processes [skinning, washing (1), eviscerating, washing (2), trimming, and pre-cooling] and from the environment (workers’ hands, slaughterhouses’ ground, and appliances) in five slaughterhouses, using PBS sampling swabs (SWAB-10 PBS, ELAB Scientific, Escondido, United States), and transported them to the laboratory at 4°C on the same day.

#### Isolation and Identification of Microorganisms

##### *Escherichia coli* Counts

The swabs collected from the five swine slaughterhouses were fully vortexed and diluted according to a gradient. Sample solutions (1 mL) of three suitable dilutions were vertically transferred into the center of the *E. coli*/Coliform Count Plate (3M Petrifilm 6414, 3M Health Care, Saint Paul, United States) using a pipette and cultured for about 24 h at 37°C. From to the instructions of 3M Petrifilm 6414, the blue and dark blue colonies with bubbles were identified as *E. coli*. The colonies with the above morphological characteristics were selected for *E. coli* count.

##### *Salmonella* Detection

Two slaughterhouses were randomly selected for isolation of *Salmonella* on swine carcass surfaces during slaughtering. The specific isolation method referred to ISO 6579-1:2017 (2017), and we used *inv*A primer for PCR identification (Rahn et al., 1992).

##### Statistical Analysis

Monitoring data of *E. coli* contamination during swine-slaughtering processes were statistically analyzed using SPSS 22 (IBM, Qingdao, China). *T*-test was used to investigate the statistical differences between adjacent processes.

#### Multilocus Sequence Typing of *Escherichia coli*

We picked out *E. coli* on the count plates and inoculated them on MacConkey culture medium (CM908, Lu Qiao Technology, Beijing, China). All strains on MacConkey medium were cultured for 18 to 24 h at 37°C and purified two times. Strains were randomly selected from all of the *E. coli* isolates for multilocus sequence typing. Seven housekeeping genes of *E. coli* (*adk, fumC, gyrB, icd, mdh, purA*, and *recA*) were amplified according to the recommended PCR amplification procedure and annealing temperature (Wirth et al., 2006). PCR-amplified products were submitted to Beijing Qingke Biotechnology Co., Ltd. for sequencing. The cut sequences were inputted into the multilocus sequence typing database,^1^ and the numerical sequence numbers of each allele was searched. The final seven sequence numbers determined the sequence type (ST) of a strain. The profile including seven gene loci of all strains was entered into BioNumerics software (v.7.6, Applied Maths, Kortrijk, Belgium) to construct the minimum spanning tree of different slaughtering processes. In the minimum spanning tree, circles correspond to STs, and the size of each circle is proportional to the number of isolates in each ST. The maximum risk process was determined according to the typing results.

### Exposure Assessment Model of *Escherichia coli*

We selected a single swine as the object for *E. coli* exposure assessment. The carcasses of swine after skinning were directly exposed to air and the environment for the subsequent processing. Therefore, we regarded skinning as the starting point of the evaluation procedure, and the downstream processes included washing (1), eviscerating, washing (2), trimming, and pre-cooling.

#### Exposure Assessment Tool

The distribution fitting function of the risk assessment software @Risk (v.7.0, Palisade, NY, United States) was applied to process the data. The parameters and variables involved in the exposure assessment were expressed by formulas, specific values, or distributions. The model was established in Excel 2007 worksheet (Microsoft, Redmond, United States), and the Latin hypercube sampling method in @Risk was used for Monte Carlo simulation during model simulation.

#### Data Fitting

This model for *E. coli* exposure assessment described the changes in *E. coli* concentration and the positive rate in a swine-slaughtering line [processes of skinning, washing (1), eviscerating, washing (2), trimming, and pre-cooling were included]. We used the monitoring data on *E. coli* prevalence rate in a swine-slaughtering line for data fitting by Fit Distribution, which formed the basis distributions in this exposure assessment model.

The data of *E. coli* concentration for quantitative fitting are shown in Supplementary Table 1. Results of *E. coli* quantitative data fitting in differently sized slaughterhouses are shown in the “Distribution” part of Table 1 and Supplementary Figures 1–5. In each slaughtering process, the minimum, maximum, mean, standard test dose (std), and deviation (dev) of *E. coli* concentration of our data were input for data fitting, and optimum distributions which could describe the variation of *E. coli* concentration in each slaughtering process were output. The quantitative data-fitting distribution of *E. coli* adopted the data-fitting function in @Risk software to obtain the best-fitting function expression. Results of *E. coli* qualitative data fitting in differently sized slaughterhouses are also shown in the “Distribution” part of Table 1. We adopted RiskDiscrete distribution to describe the changed *E. coli* prevalence at each slaughtering process: RiskDiscrete ({0, 1},{a, b}), where “0” represents *E. coli* negative, “1” represents *E. coli* positive, “a” is the value of the *E. coli* negative rate, and “b” is the value of the *E. coli* positive rate.

**TABLE 1:** *Escherichia coli* exposure assessment model of swine-slaughtering processes.

Swine slaughterhouses’ size	Module	Symbol	Description	Unit	Distribution/model	References
All slaughterhouses	Skinning	m	Surface area of a single pig	100cm^2^	RiskUniform (96,180)	Investigation
		Las	Log number of *E. coli* after skinning	Log_10_CFU/100cm^2^	RiskTriang (2.7825, 5.1249, 5.1249)	—
		Pas	Prevalence of *E. coli* after skinning	—	RiskDiscrete ({0,1}, {0.3750, 0.6250})	Data simulation
			Output of *E. coli* after skinning	Log_10_CFU/100cm^2^	RiskOutput (“skinning”) + IF(Pas = 0, 0, Las)	—
	Washing (1)	Lcw1	Log number of *E. coli* changed through washing (1)	Log_10_CFU/100cm^2^	RiskBetaGeneral (6.5293, 4.0775, −2.5387, 2.4624)	Data simulation
		Law1	Log number of *E. coli* after washing (1)	Log_10_CFU/100cm^2^	Las-Lcw (1)	—
		Paw1	Prevalence of *E. coli* after washing (1)	—	RiskDiscrete ({0,1}, {0.4500, 0.5500})	Data simulation
			Output of *E. coli* after washing(1)	Log_10_CFU/100cm^2^	RiskOutput [“washing (1)”] + IF [Paw (1) = 0, 0, Law (1)]	—
	Eviscerating	Lce	Log number of *E. coli* changed through eviscerating	Log_10_CFU/100cm^2^	RiskBetaGeneral (13.124, 10.199, −5.0443, 5.8443)	Data simulation
		Lae	Log number of *E. coli* after eviscerating	Log_10_CFU/100cm^2^	Law (1) + Lce	—
		Pae	Prevalence of *E. coli* after eviscerating	—	RiskDiscrete ({0, 1}, {0.4182, 0.5818})	Data simulation
			Output of *E. coli* after eviscerating	Log_10_CFU/100cm^2^	RiskOutput (“eviscerating”) + IF (Pae = 0, 0, Lae)	—
	Washing (2)	Lcw2	Log number of *E. coli* changed through washing (2)	Log_10_CFU/100cm^2^	RiskBetaGeneral (26.609, 19.752, −11.782, 10.133)	Data simulation
		Law2	Log number of *E. coli* after washing (2)	Log_10_CFU/100cm^2^	Lae-Lcw (2)	—
		Paw2	Prevalence of *E*. coli after washing (2)	—	RiskDiscrete ({0, 1}, {0.6182, 0.3818})	Data simulation
			Output of *E. coli* after washing (2)	Log_10_CFU/100cm^2^	RiskOutput [“washing (2)”] + IF [Paw (2) = 0, 0, Law (2)]	—
	Trimming	Lct	Log number of *E. coli* changed through trimming	Log_10_CFU/100cm^2^	RiskBetaGeneral (33.414, 29.533, −17.982, 16.126)	Data simulation
		Lat	Log number of *E. coli* after trimming	Log_10_CFU/100cm^2^	Law (2) + Lct	—
		Pat	Prevalence of *E*. coli after trimming	—	RiskDiscrete ({0, 1}, {0.149, 0.851})	Data simulation
			Output of *E. coli* after trimming	Log_10_CFU/100cm^2^	RiskOutput (“trimming”) + IF (Pat = 0, 0, Lat)	—
	Pre-cooling	Lcp	Log number of *E. coli* changed through pre-cooling	Log_10_CFU/100cm^2^	RiskNormal (0.75094, 3.1227)	Data simulation
		Lap	Log number of *E. coli* after pre-cooling	Log_10_CFU/100cm^2^	Lat-Lcp	—
		Pap	Prevalence of *E. coli* after pre-cooling	—	RiskDiscrete ({0, 1}, {0.3263, 0.6737})	Data simulation
			Output of *E. coli* after pre-cooling	Log_10_CFU/100cm^2^	RiskOutput (“pre-cooling”) + IF (Pap = 0, 0, Lap)	—
Big slaughterhouses	Skinning	m	Surface area of a single pig	100cm^2^	RiskUniform (96, 180)	Investigation
		Las	Log number of *E. coli* after skinning	Log_10_CFU/100cm^2^	RiskUniform (2.8672, 5.2577)	—
		Pas	Prevalence of *E*. coli after skinning	—	RiskDiscrete ({0, 1}, {0.4000, 0.6000})	Data simulation
			Output of *E. coli* after skinning	Log_10_CFU/100cm^2^	RiskOutput (“skinning”) + IF (Pas = 0, 0, Las)	—
	Washing (1)	Lcw (1)	Log number of *E. coli* changed through washing (1)	Log_10_CFU/100cm^2^	RiskBetaGeneral (6.4614, 6.2514, −2.7547, 3.3950)	Data simulation
		Law (1)	Log number of *E. coli* after washing (1)	Log_10_CFU/100cm^2^	Las-Lcw (1)	—
		Paw (1)	Prevalence of *E. coli* after washing (1)	—	RiskDiscrete ({0, 1}, {0.4000, 0.6000})	Data simulation
			Output of *E. coli* after washing (1)	Log_10_CFU/100cm^2^	RiskOutput [“washing (1)”] + IF [Paw (1) = 0, 0, Law (1)]	—
	Eviscerating	Lce	Log number of *E. coli* changed through eviscerating	Log_10_CFU/100cm^2^	RiskBetaGeneral (8.0576, 7.9915, −4.4410, 6.2900)	Data simulation
		Lae	Log number of *E. coli* after eviscerating	Log_10_CFU/100cm^2^	Law (1) + Lce	—
		Pae	Prevalence of *E. coli* after eviscerating	—	RiskDiscrete ({0, 1}, {0.5143, 0.4857})	Data simulation
			Output of *E. coli* after eviscerating	Log_10_CFU/100cm^2^	RiskOutput (“eviscerating”) + IF (Pac = 0, 0, Lae)	—
	Washing (2)	Lcw (2)	Log number of *E. coli* changed through washing (2)	Log_10_CFU/100cm^2^	RiskBetaGeneral (16.939, 17.633, −9.6107, 11.382)	Data simulation
		Law (2)	Log number of *E. coli* after washing (2)	Log_10_CFU/100cm^2^	Lae-Lcw (2)	—
		Paw (2)	Prevalence of *E*. coli after washing (2)	—	RiskDiscrete ({0, 1}, {0.6857, 0.3143})	Data simulation
			Output of *E. coli* after washing (2)	Log_10_CFU/100cm^2^	RiskOutput [“washing (2)”] + IF [Paw (2) = 0, 0, Law (2)]	—
	Trimming	Lct	Log number of *E. coli* changed through trimming	Log_10_CFU/100cm^2^	RiskNormal (0.22194, 2.4674)	Data simulation
		Lat	Log number of *E. coli* after trimming	Log_10_CFU/100cm^2^	Law (2) + Lct	—
		Pat	Prevalence of *E. coli* after trimming	—	RiskDiscrete ({0, 1}, {0.133, 0.867})	Data simulation
			Output of *E. coli* after trimming	Log_10_CFU/100cm^2^	RiskOutput (“trimming”) + IF (Pat = 0, 0, Lat)	—
	Pre-cooling	Lcp	Log number of *E. coli* changed through pre-cooling	Log_10_CFU/100cm^2^	RiskNormal (1.0892, 3.6776)	Data simulation
		Lap	Log number of *E. coli* after pre-cooling	Log_10_CFU/100cm^2^	Law (2)-Lcp	—
		Pap	Prevalence of *E. coli* after pre-cooling	—	RiskDiscrete ({0, 1}, {0.2667, 0.7333})	Data simulation
			Output of *E. coli* after pre-cooling	Log_10_CFU/100cm^2^	RiskOutput (“pre-cooling”) + IF (Pap = 0, 0, Lap)	—
Small slaughterhouses	Skinning	m	Surface area of a single pig	100cm^2^	RiskUniform (96, 180)	Investigation
		Las	Log number of *E. coli* after skinning	Log_10_CFU/100cm^2^	RiskUniform (4.0586, 5.1097)	—
		Pas	Prevalence of *E. coli* after skinning	—	RiskDiscrete ({0, 1}, {0.3500, 0.6500})	Data simulation
			Output of *E. coli* after skinning	Log_10_CFU/100cm^2^	RiskOutput (“skinning”) + IF (Pas = 0, 0, Las)	—
	Washing (1)	Lcw (1)	Log number of *E. coli* changed through washing (1)	Log_10_CFU/100cm^2^	RiskTriang (-0.65974, 0.53949, 1.7135)	Data simulation
		Law (1)	Log number of *E. coli* after washing (1)	Log_10_CFU/100cm^2^	Las-Lcw (1)	—
		Paw (1)	Prevalence of *E. coli* after washing (1)	—	RiskDiscrete ({0, 1}, {0.5000, 0.5000})	Data simulation
			Output of *E. coli* after washing (1)	Log_10_CFU/100cm^2^	RiskOutput [“washing (1)”] + IF [Paw (1) = 0, 0, Law (1)]	—
	Eviscerating	Lce	Log number of *E. coli* changed through eviscerating	Log_10_CFU/100cm^2^	RiskBetaGeneral (6.8886, 7.0685, −1.3571, 3.7428)	Data simulation
		Lae	Log number of *E. coli* after eviscerating	Log_10_CFU/100cm^2^	Law (1) + Lce	—
		Pae	Prevalence of *E. coli* after eviscerating	—	RiskDiscrete ({0, 1}, {0.2500, 0.7500})	Data simulation
			Output of *E. coli* after eviscerating	Log_10_CFU/100cm^2^	RiskOutput (“eviscerating”) + IF (Pac = 0, 0, Lae)	—
	Washing (2)	Lcw (2)	Log number of *E. coli* changed through washing (2)	Log_10_CFU/100cm^2^	RiskBetaGeneral (22.859, 20.081, −6.4813, 7.6571)	Data simulation
		Law (2)	Log number of *E. coli* after washing (2)	Log_10_CFU/100cm^2^	Lae-Lcw (2)	—
		Paw (2)	Prevalence of *E. coli* after washing (2)	—	RiskDiscrete ({0, 1}, {0.5000, 0.5000})	Data simulation
			Output of *E. coli* after washing (2)	Log_10_CFU/100cm^2^	RiskOutput [“washing (2)”] + IF [Paw (2) = 0, 0, Law (2)]	—
	Trimming	Lct	Log number of *E. coli* changed through trimming	Log_10_CFU/100cm^2^	RiskBetaGeneral (18.039, 16.645, −8.3443, 8.3145)	Data simulation
		Lat	Log number of *E. coli* after trimming	Log_10_CFU/100cm^2^	Law (2) + Lct	—
		Pat	Prevalence of *E. coli* after trimming	—	RiskDiscrete ({0, 1}, {0.214, 0.786})	Data simulation
			Output of *E. coli* after trimming	Log_10_CFU/100cm^2^	RiskOutput (“trimming”) + IF (Pat = 0, 0, Lat)	—
	Pre-cooling	Lcp	Log number of *E. coli* changed through pre-cooling	Log_10_CFU/100cm^2^	RiskNormal (0.12818, 1.9733)	Data simulation
		Lap	Log number of *E. coli* after pre-cooling	Log_10_CFU/100cm^2^	Law (2)-Lcp	—
		Pap	Prevalence of *E. coli* after pre-cooling	—	RiskDiscrete ({0, 1}, {0.5500, 0.4500})	Data simulation
			Output of *E. coli* after pre-cooling	Log_10_CFU/100cm^2^	RiskOutput (“pre-cooling”) + IF (Pap = 0, 0, Lap)	—

#### Monte Carlo Simulation and Model Establishment

On the basis of the fitting functions generated above, an exposure assessment model was established by @Risk and Monte Carlo simulation. In the logic relationship of this *E. coli* exposure assessment model, the output of the previous process was set as the input of the next process. The data of samples after skinning were set as the initial contamination load, and then the *E. coli* concentration or prevalence of other slaughtering processes was outputted successively through the Monte Carlo simulation. The number of iterations per simulation calculation was 10, 000 (Huang et al., 2017b). The system extracted a value from the distribution of each input variable to complete each iterative computation randomly.

#### Sensitivity Analysis

Through the correlation coefficient of model fitting, the correlation between *E. coli* contamination in pork after pre-cooling and after each slaughtering process was analyzed to determine the risk contribution of each process to *E. coli* contamination in terminal pork products. Sensitivity analyses were performed in @Risk software. The Spearman level correlation coefficient was calculated to be between −1 and +1, where + and - denote positive correlation and negative correlation, respectively. The positive correlation coefficient indicated that the process had a risk elimination effect, while the negative correlation coefficient indicated that the process had a risk introduction effect. The higher the absolute value of the correlation coefficient, the greater the impact of this slaughtering process on the risk posed by terminal pork products.

## Results

### Monitoring Data of *Escherichia coli* Contamination During Swine-Slaughtering Processes

**FIGURE 1:**
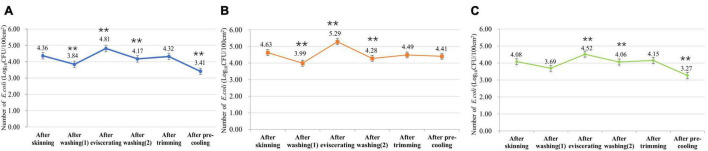
*Escherichia coli* contamination of surfaces of swine carcasses in slaughterhouses of different sizes. **(A)** Number of *E. coli* in all swine slaughterhouses. **(B)** Number of *E. coli* in small swine slaughterhouses. **(C)** Number of *E. coli* in big swine slaughterhouses. **means the statistical difference between this process and the previous process is extremely significant (*P* < 0.01).

From the *E. coli* monitoring data of slaughtering processes in slaughterhouses of different sizes (Figure 1), it can be found that the contamination of *E. coli* decreased significantly after washing and pre-cooling (*P* < 0.01). The number of *E. coli* in slaughterhouses of different sizes increased significantly after eviscerating (*P* < 0.01), and it had particularly obvious impact on small slaughterhouses. The number of *E. coli* after trimming in slaughterhouses of different sizes also increased. In addition, the overall *E. coli* contamination of carcass swabs in big slaughterhouses was lower than that in small slaughterhouses, indicating that the sanitary control of large slaughterhouses was better.

### Establishment of an *Escherichia coli* Exposure Assessment Model

#### Model Simulation of *Escherichia coli* Contamination on the Swine Carcass Surface After Pre-cooling

**FIGURE 2:**
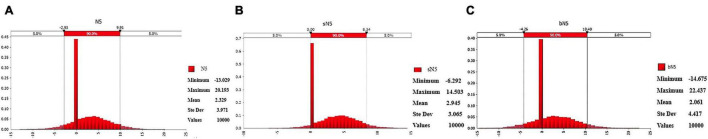
Probability distribution of *Escherichia coli* contamination of swine carcasses after pre-cooling in slaughterhouses of different sizes. **(A)** Probability distribution of *E. coli* contamination of swine carcasses after pre-cooling in all swine slaughterhouses. **(B)** Probability distribution of *E. coli* contamination of swine carcasses after pre-cooling in small swine slaughterhouses. **(C)** Probability distribution of *E. coli* contamination of swine carcasses after pre-cooling in big swine slaughterhouses. N5- Log number of *E. coli* on swine carcasses after pre-cooling.

Through the established model for slaughterhouses of different sizes, we showed that 90% of the *E. coli* contamination of a single pig in big slaughterhouses after skinning, washing (1), eviscerating, washing (2), trimming, and pre-cooling may be distributed between −4.26 and 10.40 log_10_ CFU/100 cm^2^ (Figure 2C), with an average of 2.06 log_10_ CFU/100 cm^2^. After the above processes, 90% of the *E. coli* contamination of a single pig in a small slaughterhouse may be distributed between 0.00 and 8.34 log_10_ CFU/100 cm^2^ (Figure 2B), with an average of 2.95 log_10_ CFU/100 cm^2^. In general, 90% of the *E. coli* contamination of a single pig after the above processes may be distributed between −2.93 and 9.91 log_10_ CFU/100 cm^2^ (Figure 2A), with an average of 2.33 log_10_ CFU/100 cm^2^.

#### *Escherichia coli* Contamination Pattern During Slaughtering Simulated by the Model

**FIGURE 3:**
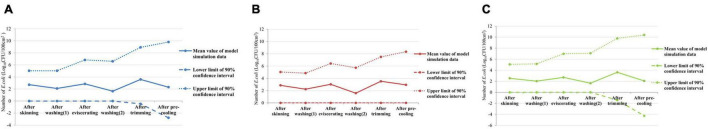
*Escherichia coli* contamination simulated by exposure model of slaughterhouses of different sizes. **(A)** *E. coli* contamination simulated by exposure model of all slaughterhouses. **(B)** *E. coli* contamination simulated by exposure model of small swine slaughterhouses. **(C)** *E. coli* contamination simulated by exposure model of big swine slaughterhouses.

Through the established exposure assessment model, the total number of *E. coli* on the surface of swine carcasses in skinning, washing (1), eviscerating, washing (2), trimming, and pre-cooling were further simulated. According to the mean value obtained, the *E. coli* contamination pattern on the surfaces of swine carcasses during slaughtering in slaughterhouses of different sizes were established (Figure 3). In general, regardless of slaughterhouse size, the number of *E. coli* after washing decreased to some extent, indicating that the washing process can effectively flush out some *E. coli*. The number of *E. coli* increased obviously after trimming, while it decreased after pre-cooling, especially in big slaughterhouses. Although the *E. coli* contamination pattern of the whole slaughtering processes in large and small slaughterhouses were the same, the *E. coli* contamination of small slaughterhouses was slightly higher than that of large slaughterhouses, indicating that the overall hygiene condition of large slaughterhouses was better. In addition, by comparing the *E. coli* monitoring data of different slaughterhouses (Figure 1) with the results of the established model (Figure 3), we found that the actual monitoring data fell within the 90% confidence interval of the model simulation results. This indicates that the credibility of the model was quite good.

#### Analysis of Key Control Points of Microbial Contamination Risk

**FIGURE 4:**
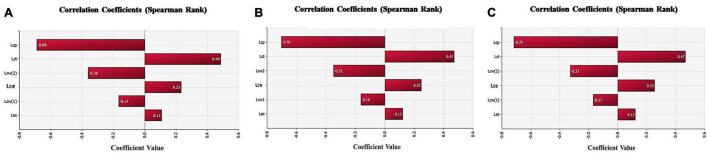
Sensitivity analysis of each slaughtering processes in the model. **(A)** Sensitivity analysis of each slaughtering process in all swine slaughterhouses. **(B)** Sensitivity analysis of each slaughtering process in small swine slaughterhouses. **(C)** Sensitivity analysis of each slaughtering process in big swine slaughterhouses. Lcp-Log number of *Escherichia coli* changed through pre-cooling;Lct-Log number of *E. coli* changed through trimming;Lcw-Log number of *E. coli* changed through washing;Lce- Log number of *E. coli* changed through eviscerating;Las- Log number of *E. coli* after skinning.

The correlation between the *E. coli* contamination of the surface of terminal swine carcasses and the slaughtering processes were discussed through sensitivity analysis of the parameters in the exposure assessment model. The risk contribution of each slaughtering process to the *E. coli* contamination of the surface of terminal swine carcasses can be determined. As shown in Figure 4, the sensitivity analysis in slaughterhouses of different sizes showed that the trimming process contributed the most to the risk of *E. coli* contamination (correlation coefficient of all slaughterhouses, small slaughterhouses and large slaughterhouses were 0.49, 0.47, 0.47, respectively), followed by the eviscerating process (correlation coefficient of all slaughterhouses, small slaughterhouses and large slaughterhouses were 0.23, 0.25, 0.25, respectively). Therefore, the trimming process was the most important key control point affecting the *E. coli* contamination on the surfaces of terminal swine carcasses, followed by the eviscerating process. The pre-cooling and washing processes were negatively correlated, indicating that they had a positive effect on reducing the number of *E. coli*.

The skinning process had a slight risk introduction effect to the *E. coli* contamination of the surface of terminal swine carcasses.

### Further Validation of the Exposure Assessment Model

#### Contamination State of *Salmonella* in the Slaughtering Processes

**FIGURE 5:**
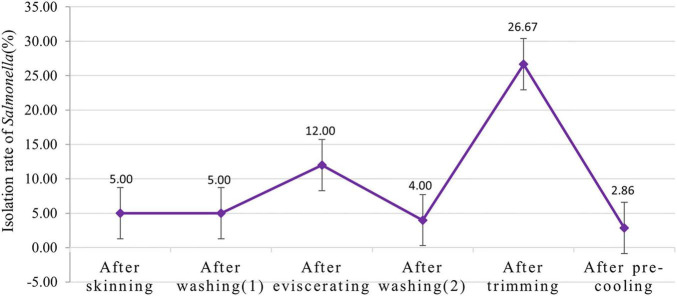
Isolation rate of *Salmonella* in different slaughtering processes.

Two slaughterhouses were randomly selected to isolate and identify *Salmonella* on the surface of swine carcasses, and then the *Salmonella* isolation rate in the different slaughtering processes were analyzed (Figure 5). It can be seen from Figure 5 that the *Salmonella* isolation rate increased from 4.00% to the maximum of 26.67% after trimming. Therefore, the contamination state of *Salmonella* in different slaughtering processes can support the most important key control point obtained by the exposure assessment model.

#### Results of Multilocus Sequence Typing of *Escherichia coli*

**FIGURE 6:**
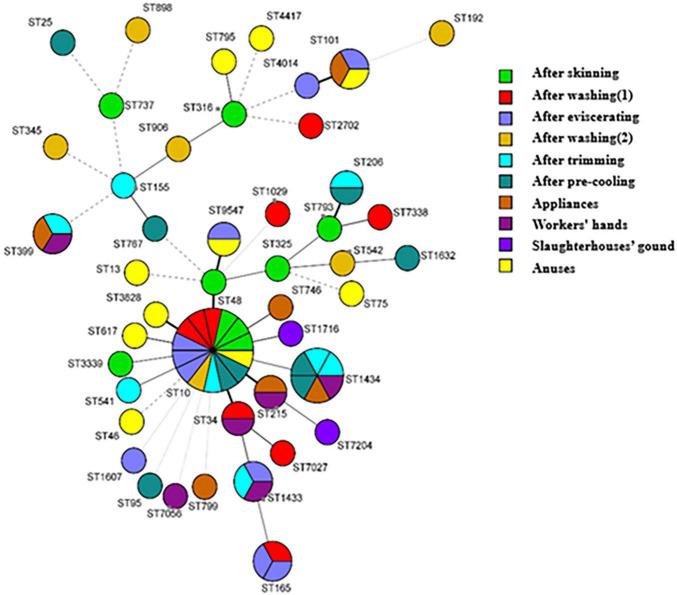
The minimum spanning tree of 51 strains of *Escherichia coli*.

The typing results show that there were various sequence types of *E. coli* in the whole swine-slaughtering chain; 51 *E. coli* strains were divided into 32 ST types. The specific typing results are shown in Supplementary Table 2. The minimum spanning tree (Figure 6) shows that the most dominant ST type was ST10, and that ST10 strains could be isolated from all slaughtering processes. The ST10 strain was also presented in anal swabs, indicating that ST10 was introduced by the process of swine breeding. The dominant type in skinning, washing (1), eviscerating, and washing (2) processes is ST10. ST1434 began to appear in the trimming process and then became the dominant type in the trimming and pre-cooling processes. It is worth noting that ST1434 also included samples from workers’ hands and appliances. Therefore, the trimming process can be regarded as the most important risk process, which further proved the most important key control point obtained by the exposure assessment model.

## Discussion

Risk assessments of food safety are an essential part of the risk analysis process (FAO/WHO, 2005). Microbial risk assessments of livestock and poultry products are of great significance to improve the quality and safety risk management system of livestock and poultry products, provide high-quality livestock and poultry products for consumers, and guide safety in enterprises’ production. In China, most published studies on microbiological risk assessments are qualitative, describing the risk characterization of pathogens in specific foods (Huang et al., 2017b). In addition, risk assessments in China are mostly conducted for pathogenic microorganisms in livestock and poultry products. To our knowledge, there are no studies on establishing models and carrying out risk assessments through data monitoring for contamination using health-indicator bacteria. In other countries, microbial risk assessments of livestock and poultry products have focused on consumption risks (Fares and Rouviere, 2010) or the exposure assessment of chemical hazards and certain toxins in livestock and poultry products (Flores et al., 2019; Sanchez-Montero et al., 2019). Therefore, the *E. coli* exposure assessment model established in this study not only fills the gaps in microbial exposure assessment of swine slaughtering chain to a certain extent, but also further scientifically supplements the HACCP system of livestock and poultry products in China.

The detection level of *E. coli*, a health-indicator bacterium, can reflect the potential food risk caused by pathogenic bacteria. Therefore, the risk assessment of *E. coli* can strengthen food hygiene control and reduce the risk of disease. In this study, skinning, washing (1), eviscerating, washing (2), trimming, and pre-cooling processes were successively incorporated into the establishment of the exposure assessment model of *E. coli* contamination. The model was used to simulate the *E. coli* contamination on the surface of swine carcasses in differently sized slaughterhouses, and then the contamination pattern of *E. coli* was constructed. We concluded that the contamination level of *E. coli* in the whole slaughtering process of big slaughterhouses is lower than in small slaughterhouses. Therefore, we can illustrate to a certain extent that the health control of big slaughterhouses is better than that of small slaughterhouses. This conclusion is consistent with the monitoring conclusion for *E. coli* in pork by the USDA (United States Department of Agriculture, 2011). Because of the influence of water activity and low temperature of swine carcasses, the pre-cooling process significantly reduced the *E. coli* contamination of the swine carcass surface, especially for big slaughterhouses. The contamination of *E. coli* increased to the maximum after eviscerating, which may be caused by cross-contamination from a broken gut or the environment.

According to the sensitivity analysis of the model, the most important key control point is the trimming process whether in big or small slaughterhouses. ST1434 appeared in the carcass swabs of the trimming process and then became the dominant type in this process. ST1434 also existed in the workers’ hands and appliances swabs, illustrating that there was cross-contamination between carcasses and the environment in the trimming process. The contamination carried by environmental factors such as the workers’ hands or appliances in the trimming process, spreading downstream along the slaughtering chain. Therefore, the trimming process can be considered as an important risk process. The eviscerating process is another key control point; the reason for the increase of *E. coli* contamination in this process may be the eviscerating operation, which led to visceral rupture and overflow of autologous microorganisms (Huang et al., 2017a; Crotta et al., 2018) and then contaminated appliances and workers’ hands and caused cross-contamination. In addition, according to the typing results, ST1433 and ST101 in the eviscerating process also existed in the workers’ hands and appliances. Therefore, it can be reasonably speculated that *E. coli* in the environment is closely related to *E. coli* on carcasses in this process. The minimum spanning tree showed that ST10 strains could be isolated from anal swabs and all slaughtering processes, which indicated that the ST10 was widespread in the slaughtering chain and was more likely to be carried by the pigs themselves and spread along the slaughtering chain.

The key control points of swine-slaughtering processes in this study were different from those of other studies, which mainly because the different slaughterhouses chosen for the study and different slaughtering processes. In fact, skinning, eviscerating, trimming, and cutting may all be the key control points for microbial contamination. In addition, the key control points of differently sized slaughterhouses in this study were the same, which maybe because the slaughtering processes of differently sized slaughterhouses in Shandong Province is basically the same, and the appliances and sanitary control measures adopted in slaughtering were also roughly similar. Using the quantitative risk assessment model of *Salmonella* contamination during swine slaughtering, Zhao et al. (2018) obtained three main key control points, which were splitting, eviscerating, and scalding; splitting was also a part of the trimming process. Pearce et al. (2004) discussed the impact of pig slaughter processes on carcass microbiology and their potential use as critical control points (C) during pork production. The main critical control points they concluded were bleeding and eviscerating. Yu et al. (1999) investigated swine slaughtering operations in America to establish their critical control points. Their study indicated that the polishing and eviscerating processes can be identified as critical control points. Liu et al. (2013) applied the HACCP principle to analyze the microbial hazards in chilled-pork processing. The main key control points they obtained were bleeding, eviscerating, pre-cooling, cutting, and packaging, of which the cutting process was the most important key control point. Our study did not involve the cutting process, but the trimming and cutting processes used appliances most frequently. Zhou et al. (2018) considered that eviscerating and polishing were the main key risk points in the study on the key risk points of *Salmonella* during swine slaughtering. Although all kinds of key control points for pathogenic microorganism risk assessments in swine-slaughtering process were different, the eviscerating process and the processes with frequent use of appliances were often regarded as the key control points in slaughtering (Arguello et al., 2013). This is consistent with the conclusion we obtained, that is, that the trimming process is the most important key control point, and the eviscerating process is the second.

The results of *E. coli* multilocus sequence typing and the *Salmonella* contamination monitoring data show that the trimming process was the most important key control point, the same as the conclusion from the exposure assessment model. The credibility of the constructed exposure assessment model was verified. Notably, from the actual monitoring data of *E. coli* during slaughtering in differently sized slaughterhouses, it can be found that *E. coli* contamination is the most serious in the eviscerating process, but the most important key control point of the exposure assessment model is the trimming process. We speculated that this may be because the monitoring data adopted the commonly used deterministic numerical calculation method such as the mean value. However, the model simulation was a statistical analysis of a large number of sample values after thousands of simulations; the results that met a certain accuracy were then obtained. Therefore, the exposure assessment model has more scientific mathematical basis and statistical significance.

Of course, there are some uncertainties in the exposure evaluation of microorganisms due to various factors. The uncertainties of the evaluation model established in this study include firstly the uncertainty of the process and model. The study assumed that *E. coli* did not proliferate throughout the slaughtering processes, but *E. coli* can proliferate in the actual processes. Second is the uncertainty of slaughtering modes in different slaughterhouses. The model was based on the slaughtering modes of several representative enterprises in Shandong; the slaughtering processes of different enterprises are different, however, because of regional or policy factors. Therefore, the model is not necessarily applicable to slaughterhouses with different slaughtering modes. The uncertainties of the model and the complexities of microorganisms affect the accuracy and effectiveness of the final evaluation results.

In this study, an exposure assessment model of *E. coli* contamination suitable for the general swine-slaughtering process in China was established. The results of correlation analysis showed that the trimming process is the most important key control point, which can be further proved from different aspects by using the contamination state of *Salmonella* and the multilocus sequence typing results of *E. coli*. Therefore, the sanitation control of workers’ hands in the trimming process should be strengthened in a more targeted manner to reduce potential cross-contamination as much as possible. Although this model contained limitations and assumptions, as with all QMRA, it provides a scientific basis for microbial hygiene supervision during swine slaughtering, as well as technical support for the prevention and control of pork-derived foodborne diseases in the future.

## Data Availability Statement

The original contributions presented in the study are included in the article/Supplementary Material, further inquiries can be directed to the corresponding authors.

## Author Contributions

TY was involved in microbial and molecular experiment, data analysis, investigation, and writing—original draft. GZ contributed to conception and design of the study, model construction, data curation, and writing—review and editing. YL, LW, YG, JZ, NL, XH, QZ, JL, and XZ performed investigation. JW was involved in project administration, funding acquisition, and supervision. YX done project administration and supervision. All authors contributed to the article and approved the submitted version.

## Conflict of Interest

The authors declare that the research was conducted in the absence of any commercial or financial relationships that could be construed as a potential conflict of interest.

## Publisher’s Note

All claims expressed in this article are solely those of the authors and do not necessarily represent those of their affiliated organizations, or those of the publisher, the editors and the reviewers. Any product that may be evaluated in this article, or claim that may be made by its manufacturer, is not guaranteed or endorsed by the publisher.

## Footnotes

1
http://mlst.warwick.ac.uk/mlst/mlst/dbs/Ecoli
