# Supplementary material for: Analysis of Key Control Points of Microbial Contamination Risk in Pork Production Processes Using a Quantitative Exposure Assessment Model
Source: Front Microbiol. 2022 Mar 24;13:828279. doi: 10.3389/fmicb.2022.828279 (PMC8992707; doi:10.3389/fmicb.2022.828279)
Supplement: Supplementary file 1 [file Image_1.PDF]

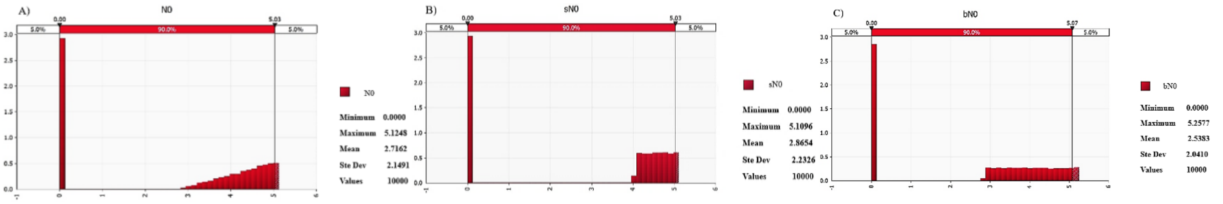

FIGURE S1 Probability distribution of *E. coli* contamination on swine carcasses after skinning in slaughterhouses of different sizes.

(A) in all swine slaughterhouses. **(B)** in small swine slaughterhouses. **(C)** in big swine slaughterhouses.

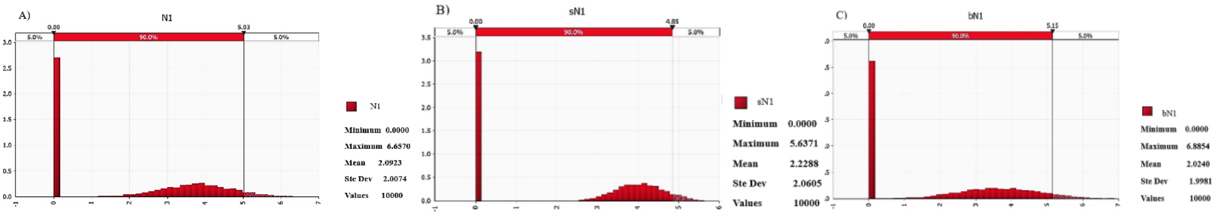

FIGURE S2 Probability distribution of *E. coli* contamination on swine carcasses after washing (1) in slaughterhouses of different sizes.

(A) in all swine slaughterhouses. **(B)** in small swine slaughterhouses. **(C)** in big swine slaughterhouses.

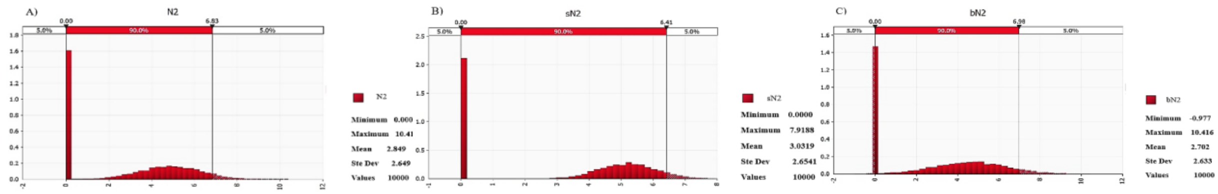

FIGURE S3 Probability distribution of *E. coli* contamination on swine carcasses after eviscerating in slaughterhouses of different sizes.

(A) in all swine slaughterhouses. **(B)** in small swine slaughterhouses. **(C)** in big swine slaughterhouses.

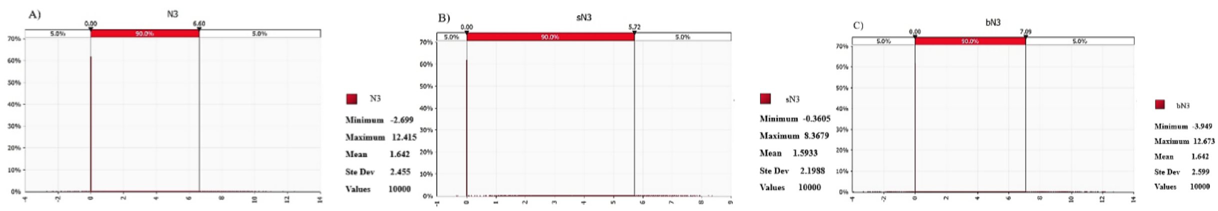

FIGURE S4 Probability distribution of *E. coli* contamination on swine carcasses after washing (2) in slaughterhouses of different sizes.

(A) in all swine slaughterhouses. **(B)** in small swine slaughterhouses. **(C)** in big swine slaughterhouses.

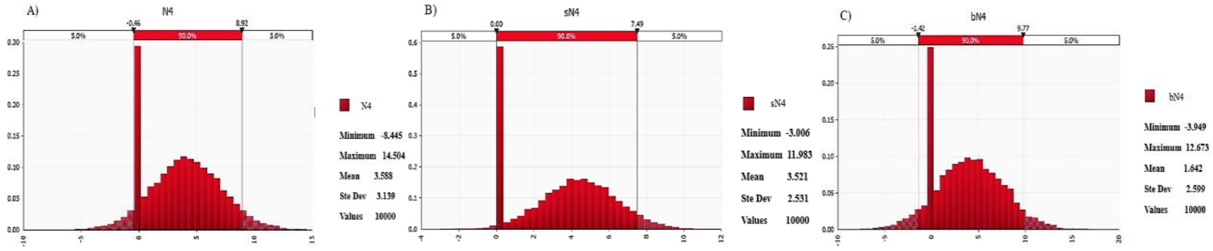

FIGURE S5 Probability distribution of *E. coli* contamination on swine carcasses after trimming in slaughterhouses of different sizes.

(A) in all swine slaughterhouses. **(B)** in small swine slaughterhouses. **(C)** in big swine slaughterhouses.
