# Supplementary material for: Analysis of Key Control Points of Microbial Contamination Risk in Pork Production Processes Using a Quantitative Exposure Assessment Model
Source: Front Microbiol. 2022 Mar 24;13:828279. doi: 10.3389/fmicb.2022.828279 (PMC8992707; doi:10.3389/fmicb.2022.828279)
Supplement: Supplementary file 2 [file Table_1.DOCX]

| **TABLE S1** Data of *E. coli* count for model establishment (log_10_ CFU/100 cm^2^). | | | | | | | |
| --- | --- | --- | --- | --- | --- | --- | --- |
| **After skinning** | **After washing (1)** | **After eviscerating** | **After washing (2)** | | **After trimming** | | **After pre-cooling** |
| 4.00 | 3.78 | 4.37 | 4.00 | 3.22 | | 3.37 | |
| 5.00 | 3.90 | 4.43 | 4.30 | 3.64 | | 3.30 | |
| 5.12 | 3.73 | 4.12 | 3.52 | 4.00 | | 3.12 | |
| 5.05 | 4.22 | 4.56 | 3.60 | 4.32 | | 3.12 | |
| 4.20 | 3.22 | 4.48 | 4.30 | 3.95 | | 3.12 | |
| 4.87 | 4.70 | 4.12 | 3.82 | 4.21 | | 2.82 | |
| 4.99 | 3.67 | 3.82 | 3.43 | 3.22 | | 2.52 | |
| 4.64 | 4.37 | 4.30 | 4.30 | 4.60 | | 3.30 | |
| 3.85 | 4.48 | 4.30 | 4.37 | 4.37 | | 3.00 | |
| 3.00 | 3.90 | 4.22 | 3.12 | 4.48 | | 1.82 | |
| 3.64 | 3.30 | 4.85 | 4.30 | 3.78 | | 3.56 | |
| 3.60 | 3.43 | 5.05 | 5.48 | 3.99 | | 4.12 | |
| 3.56 | 3.56 | 5.78 | 3.82 | 3.56 | | 4.48 | |
| 3.52 | 3.82 | 5.10 | 4.70 | 4.85 | | 4.00 | |
| 3.85 | 3.92 | 5.48 | 5.43 | 3.95 | | 3.52 | |
| 3.12 | 2.99 | 5.43 | 4.52 | 4.73 | | 3.37 | |
| 3.43 | 3.52 | 4.94 | 4.37 | 4.60 | | 3.00 | |
| 4.24 | 3.60 | 5.75 | 5.22 | 4.40 | | 4.48 | |
| 5.05 | 2.82 | 4.82 | 5.43 | 4.44 | | 4.30 | |
| 4.87 | 3.95 | 5.64 | 4.48 | 4.64 | | 4.67 | |
| 4.01 | 3.30 | 4.90 | 4.67 | 4.62 | | 1.82 | |
| 4.88 | 3.73 | 4.80 | 5.00 | 4.67 | | 3.60 | |
| 4.11 | 3.60 | 4.43 | 4.75 | 4.41 | | 1.52 | |
| 4.92 | 3.48 | 4.56 | 4.80 | 4.49 | | 4.08 | |
| 4.70 | 3.52 | 5.43 | 3.12 | 4.46 | | 3.30 | |
| 4.12 | 3.48 | 3.64 | 4.11 | 4.53 | | 4.03 | |
| 5.05 | 3.30 | 3.70 | 4.56 | 4.61 | | 4.65 | |
| 5.12 | 4.64 | 3.56 | 3.48 | 4.68 | | 4.32 | |
| 4.60 | 4.60 | 4.90 | 3.48 | 4.68 | | 4.40 | |
| 4.67 | 4.12 | 3.70 | 3.56 | 4.41 | | 4.40 | |
| 4.30 | 4.00 | 3.97 | 3.37 | 4.54 | | 4.54 | |
| 4.48 | 4.12 | 3.52 | 3.30 | 4.08 | | 4.10 | |
| 4.75 | 4.12 | 3.73 | 3.12 | 4.14 | | 2.43 | |
| 4.52 | 4.30 | 5.03 | 3.60 | 3.73 | | 2.00 | |
| 4.22 | 4.22 | 5.56 | 3.22 | 4.42 | | 3.12 | |
| 4.94 | 4.12 | 4.73 | 4.37 | 4.19 | | 3.64 | |
| 4.85 | 3.82 | 4.94 | 5.11 | 4.07 | | 3.56 | |
|  |  | 4.88 | 3.73 | 4.03 | | 4.09 | |
|  |  | 4.85 | 3.60 | 4.14 | | 2.00 | |
|  |  | 5.45 | 4.73 | 3.56 | | 3.43 | |
|  |  | 5.21 | 4.97 | 4.01 | | 3.22 | |
|  |  | 5.00 | 4.56 | 4.07 | | 4.05 | |
|  |  | 5.12 | 4.60 | 3.67 | | 3.78 | |
|  |  | 5.60 | 5.22 | 4.03 | | 3.48 | |
|  |  | 5.43 | 4.22 | 4.15 | | 4.14 | |
|  |  | 4.48 | 4.88 | 3.99 | | 4.32 | |
|  |  | 5.64 | 4.75 | 3.88 | | 3.22 | |
|  |  | 5.30 | 4.78 | 3.73 | | 3.00 | |
|  |  | 5.48 | 5.00 | 3.78 | | 1.82 | |
|  |  | 5.64 |  | 4.82 | | 1.82 | |
|  |  | 5.70 |  | 3.60 | | 2.48 | |
|  |  | 5.67 |  | 3.67 | | 2.56 | |
|  |  |  |  | 3.48 | | 2.85 | |
|  |  |  |  | 4.04 | | 2.12 | |
|  |  |  |  | 4.64 | | 2.00 | |
|  |  |  |  | 4.22 | | 2.88 | |
|  |  |  |  | 4.73 | | 2.12 | |
|  |  |  |  | 4.67 | | 1.52 | |
|  |  |  |  | 4.70 | | 1.52 | |
|  |  |  |  | 4.37 | | 4.66 | |
|  |  |  |  | 4.22 | | 4.47 | |
|  |  |  |  | 4.75 | | 4.16 | |
|  |  |  |  | 4.56 | | 4.68 | |
|  |  |  |  | 3.88 | | 4.70 | |
|  |  |  |  | 4.43 | | 4.36 | |
|  |  |  |  | 4.27 | | 4.01 | |
|  |  |  |  | 4.32 | | 4.37 | |
|  |  |  |  |  | | 4.70 | |
|  |  |  |  |  | | 4.32 | |

The shaded part is the data of big slaughterhouses, and the rest part is the data of small slaughterhouses.

**TABLE S2** Distribution of ST types from different sources.

|  | **Sample sources** | | | **Number of**  ***E. coli* strains** | | | | **ST types** | | |
| --- | --- | --- | --- | --- | --- | --- | --- | --- | --- | --- |
| **Slaughtering processes’ swabs** | After skinning | | | 9 | | | ST10(3)/ ST3339/ ST48/ ST737/ ST316/ ST325/ ST793 | | | |
|  | After washing (1) | | | 9 | | | ST10(3)/ST34/ST7338/ST2702/ ST1029/ ST165/ ST7027 | | | |
|  | After eviscerating | | | 10 | | | ST10(3)/ ST165(2)/ ST1433/ ST101/ ST1607/ ST4014/ ST9547 | | | |
|  | After washing (2) | | | 6 | | | ST10/ ST898/ ST192/ ST345/ ST542/ ST906 | | | |
|  | After trimming | | | 8 | | | ST1434(2)/ ST1433/ST10/ ST155/ ST206/ ST399/ ST541 | | | |
|  | After pre-cooling | | | 9 | | | ST1434(2)/ST10(2) / ST25/ ST95/ ST1632/ ST206/ ST767 | | | |
|  | |  | | | |  | | | | |
| **Environmental swabs** | Appliances | | | 6 | | | ST101/ ST1434/ ST746/ ST799/ ST215/ ST399 | | | |
|  | Workers' hands | | | 6 | | | ST1433/ ST1434/ ST7056/ ST215/ ST34/ ST399 | | | |
|  | Slaughterhouses’ground | | | 2 | | | ST7204/ ST1716 | | | |
|  |  | |  | |  | | | |  |  |
| **Anal swabs** | Swines’ anuses | | | 10 | | | ST101/ST13/ST3628/ST4417/ST795/ST46/ST617/ST75/ST9547/ ST10 | | | |
